# Can early assessment of hand grip strength in older hip fracture patients predict functional outcome?

**DOI:** 10.1371/journal.pone.0213223

**Published:** 2019-08-01

**Authors:** Ivan Selakovic, Emilija Dubljanin-Raspopovic, Ljiljana Markovic-Denic, Vuk Marusic, Andja Cirkovic, Marko Kadija, Sanja Tomanovic-Vujadinovic, Goran Tulic

**Affiliations:** 1 Clinic for Physical Medicine and Rehabilitation, Clinical Center of Serbia, Belgrade, Serbia; 2 Faculty of Medicine, University of Belgrade, Belgrade, Serbia; 3 Institute of Epidemiology, Faculty of Medicine, University of Belgrade, Belgrade, Serbia; 4 Department for Medical Statistics and Informatics, Faculty of Medicine, University of Belgrade, Belgrade, Serbia; 5 Institute for Orthopaedic Surgery and Traumatology, Clinical Center of Serbia, Belgrade, Serbia; Medical University Graz, AUSTRIA

## Abstract

Decreased muscle strength is not only a risk factor for hip fracture in older patients, but plays a role in recovery of physical function. Our aim was to assess the role of grip strength measured early after hip fracture, and classified according to the EWGSOP2 criteria in predicting short- and long-term functional recovery. One hundred ninety-one patients with acute hip fracture consecutively admitted to an orthopaedic hospital have been selected. A multidimensional geriatric assessment evaluating sociodemographic variables, cognitive status, functional status and quality of life prior to fracture, as well as perioperative variables were performed. Follow-ups at 3 and 6 months after surgery were carried out to evaluate functional recovery. Multivariate regression models were used to assess the predictive role of handgrip strength. The mean age of the participants was 80.3 ±6.8 years. Thirty-five percent of our patients with clinically relevant hand grip strength weakness were significantly older, more often female, had a lower BMI, and were of worse physical health. They also had a lower cognitive level, lower Barthel index, and lower EQ5D scores before fracture. Multivariate regression analysis adjusted for age and gender revealed that hand grip weakness was an independent predictor of worse functional outcome at 3 and 6 months after hip fracture for both genders and in all age populations. Our study supports the prognostic role of hand grip strength assessed at hospital admission in patients with hip fracture. Thus, clinicians should be encouraged to include hand grip assessment in their evaluation of hip fracture patients in the acute setting in order to optimize treatment of high-risk individuals.

## Introduction

Sustaining a hip fracture is considered one of the most fatal fractures for older people that leads to impaired function, and increased morbidity and mortality, and high financial liability. These facts challenge clinicians in identifying patients at risk of worse outcome early in the course of hip fracture treatment in order to set realistic rehabilitation goals, optimize perioperative care, and define optimal rehabilitation strategies in order to reduce devastating outcomes.

Functional evaluation in patients with hip fracture is an essential part of multidimensional assessment, and has an important prognostic value. Muscle weakness is considered a key element of frailty [1] and, increasingly, of sarcopenia [2, 3]. The clinical diagnosis of sarcopenia is based on three criteria: decreased muscle mass, poor physical performance (gait speed), and decreased muscle strength [4]. The prevalence of sarcopenia in hip fracture patients ranges from 17% to 74% [5]. It is believed that sarcopenia not only enhances fracture risk, but also increases the risk of poor functional outcome after hip fracture [6–9]. Reduced muscle strength makes it more difficult to regain lost balance and decreases the mechanical loading of the skeleton leading to reduced adaptive bone remodeling [7, 10]. Generalized loss of muscle strength and muscle mass thus leads to impaired neuromuscular function and decreased mechanical loading increasing consequently the risk for both falls and fractures [7, 10].

Hand grip strength (HGS) assessment is an objective measure of overall body muscle strength and physical function [11, 12], an important measure for frailty [13], and sarcopenia [4, 14]. Various studies have shown the prognostic value of hand grip strength in patients with hip fracture [15–21]. However, very few have been carried out in the acute phase [19, 20], and none has been using the European Working Group on Sarcopenia in Older People 2 (EWGSOP2) criteria [3] to define clinically relevant hand grip weakness.

The aim of our study was to assess the EWGSOP2 threshold for grip strength assessed at admission to hospital after hip fracture to predict short- and long-term functional recovery. We hypothesized that levels of grip strength below the EWGSOP2 thresholds measured in the first 48h after hip fracture could predict an unfavorable short- and long-term functional outcome.

## Materials and methods

### Study design

All adult patients 65 years or older with an acute hip fracture who were admitted consecutively to an university associated orthopedic hospital in Serbia between March 1st 2017 and February 28th 2018 were enrolled in an open, prospective, observational cohort study. All patients with pathologic fractures, major concomitant injuries, multiple trauma, malignant diseases, imminent death as a result of an end-stage disease, inability to walk before fracture, and nonoperative treatment resulting from high surgical risk were excluded. Furthermore, patients with severe cognitive impairment, as well as patients with hand weakness as a consequence of previous neurologic disorders or hand injuries also were excluded. During the study period, 551 patients had hip fractures and were examined for eligibility. One hundred ninety-one patients were confirmed eligible and were included in the study. All patients gave written informed consent to participate in the study. The study was conducted according to the Helsinki Declaration and approved by the Ethics Committee Faculty of Medicine University of Belgrade.

### Measures

#### Baseline evaluation

We assessed all subjects through standardized patient interview with respect to sociodemographic variables (age, sex, marital status, preinjury living conditions), cognitive level, handgrip strength, prefracture functional level, and health related quality of life within 24h of admission. We also recorded perioperative variables during the primary hospital stay, such as comorbidity level, waiting time for surgery, type of fracture, surgical method, type of anesthesia, presence of postoperative complications, and length of stay (LOS).

Cognitive level was assessed with the Short Portable Mental Status Questionnaire (SPMSQ) [22]. The 10-item questionnaire classifies the patient’s cognitive level depending on the number of correct answers as lucid (8–10), mild to moderate cognitive dysfunction (3–7), and severe cognitive dysfunction (0–2). Handgrip strength was measured using a JAMAR Plus Digital Hand Dynamometer (Pennsylvania, United States). Handle position two was used for measuring grip strength. This has been assumed to be the most reliable and consistent position, and is the position advocated for routine use [23]. Patients were in the supine position, and encouraged to exhibit the greatest possible force [24]. The best recorded of 3 attempts of maximal voluntary contraction performed at 1-minute intervals of the dominant hand was considered for analysis. Hand grip strength measurements less than 16 kg in women and 27 kg in men were considered cut-points for the diagnosis of sarcopenia according to the revised EWGSOP2 criteria [3]. The pre-fracture functional status 2 weeks before hospital admission was assessed by the Barthel index [25]. The Barthel index measures performance in basic activities of daily living; its score ranges from 0 (total dependence) to 100 (total independence) [26]. General health related quality of life was measured with the EQ5D scale, which consists of a five-level response for five domains related to daily activities, mobility, self-care, usual activities, pain and discomfort, anxiety and depression [27]. Responses to the health status classification system are converted into an overall score using a published utility algorithm for the UK population [28].

We used the Charlson comorbidity index (CCI) to categorize comorbidities [29]. Patients were divided into three groups: without and mild, with CCI scores of 1–2; moderate, with CCI scores of 3–4; and severe, with CCI scores ≥5.

All patients with femoral neck fractures (84 patients (43.9%)) underwent bipolar hemiarthroplasty, whereas all patients with intertrochanteric (92 patients (48,2%)) and subtrochanteric fractures (15 patients (7.9%)) underwent open reduction and internal fixation (ORIF). In all patients early assisted ambulation was encouraged on the first postoperative day with weightbearing as tolerated, and all patients followed a standardized postoperative rehabilitation program.

#### Outcomes

**Fig 1:**
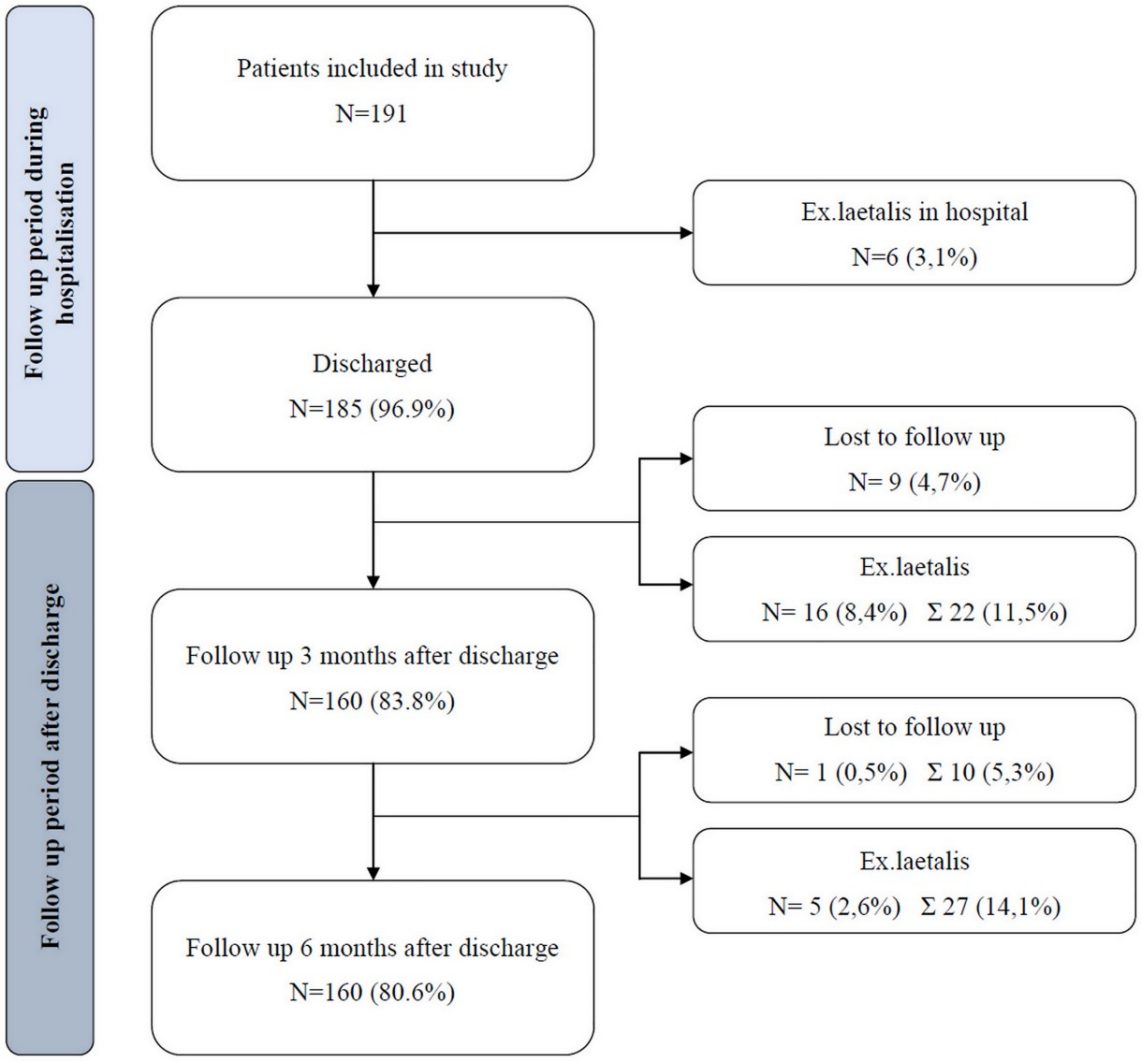
The flow of patients during the period of the investigation.

Functional status after 3 and 6 months was evaluated using the Barthel index score. The information was collected by phone interview. Data from patients who died or were lost before the first and second follow-up respectively were excluded from the study. For the analysis of Barthel index 3 months postoperatively the sample size included 160 patient (22 (11.5%) died, 9 (4.7%) were lost to follow-up). Analysis of outcomes six months after the fracture was performed on 154 patients (27 (14.1%) died, 10 (5.3%) were lost to follow-up). Fig 1 summarizes the flow of patients during the investigation period.

### Statistical analysis

Continuous variables are presented in terms of mean values with SD or median and interquartile range depending on Kolmogorov-Smirnov test of distribution normality. Categorical values are summarized as absolute frequencies and percentages. To compare patients with two different categories of grip strength a *t* test was performed for the continuous variables and a Mann-Whitney U test for ordinal variables.

In order to detect potential and independent predictors of recovery expressed as Barthel index scores after 3 and 6 months, univariate and then multivariate linear regression with collinearity diagnostic (VIF method used; variables with VIF > 4 were excluded from multivariate models) was used. Both multivariate models were adjusted for age and gender.

The significance level for all statistical tests was set at 0.05. All analyses were performed using the SPSS Inc. Released 2008. SPSS Statistics for Windows, Version 17.0. Chicago: SPSS Inc.

## Results

Our cohort consisted of 191 patients aged 66 to 97 years. The mean age was 80.3 ±6.8 years, and 77.0% of our cohort were women. The mean HGS in our cohort was 20.5 ±6.8 kg (28,7 ±6.5 kg in men, 18,1 ±4.6 kg in women). Sixty-six (34.6%) patients had clinically relevant hand grip weakness. Those patients were significantly older, more often female, had a lower BMI, and were of worse physical health. They also had a lower cognitive level, Barthel index scores and EQ5D scores before fracture. Patients with weaker grip strength were more often operated in general anesthesia (Table 1).

**Table 1:** Socio-demographic and baseline pre- and perioperative characteristics of the participants.

	Women With HGS < 16 kg Men With HGS < 27 kg N = 66 (34.6%)	Women With HGS ≥ 16 kg Men With HGS ≥ 27kg N = 125 (65.4%)	p
Age (year)*	83.53 ± 6.16	78.52 ± 6.50	<0.001
Gender**			
Male	21 (31.8%)	23 (18.4%)	0.036
Female	45 (68.2%)	102 (81.6%)	
Marital status**			
Other	40 (61.5%)	76 (61.8%)	0.973
Married	25 (38.5%)	47 (38.2%)	
Pre-injury residence**			
Home (live alone)	14 (21.2%)	31 (24.8%)	0.710
Home (live with family)	50 (75.8%)	92 (73.6%)	
Institution	2 (3.0%)	2 (1.6%)	
BMI*	23.82 ± 4.77	25.60 ± 3.91	0.008
CCI groups**			
No comorbidity/mild	21 (31.8%)	68 (54.4%)	0.009
Moderate	36 (54.6%)	42 (33.6%)	
Severe	9 (13.6%)	15 (12.0%)	
SPMSQ*	6.79 ± 1.67	7.83 ± 1.63	<0.001
EQ5D before fracture*	0.73 ± 0.17	0.83 ± 0.14	<0.001
Barthel index before fracture*	92.95 ± 7.70	96.16 ± 5.54	0.003
Type of fracture**			
Femoral neck	25 (37.9%)	59 (47.2%)	0.462
Intertrochanteric	35 (53.0%)	57 (45.6%)	
Subtrochanteric	6 (9.1%)	9 (7.2%)	
Time from admission to operation*	6.26 ± 3.17	5.75 ± 2.98	0.277
Lenght of hospital stay*	15.91 ± 5.20	16.03 ± 4.42	0.864
Surgical procedure**			
Arthroplasty	27 (36.4%)	57 (45.6%)	0.219
ORIF	39 (63.6%)	68 (54.4%)	
Type of anesthesia**			
General	51 (79.7%)	78 (64.5%)	0.032
Regional	13 (20.3%)	43 (35.5%)	
Duration of anesthesia*	118.47 ± 39.54	114.70 ± 27.18	0.504
Complications**			
Yes	18 (27.3%)	30 (24.0%)	0.620
No	48 (72.7%)	95 (76.0%)	

*Values are given as the mean with the standard deviation in parentheses

** Values are given as the number of patients with the percentage in parentheses

RR—relative risk; BMI—body mass index; CCI—Charlson Comorbidity Index; SPMSQ—Short Portable Mental Status Questionnaire; HGS—handgrip strength

Patients with relevant hand grip weakness achieved statistically significant lower Barthel index scores 3 (56.30 ±25.87 vs. 75.77 ±21.49) (Table 2) and 6 months (67.77 ±29.15 vs. 87.66 ±19.30) (Table 3) after hip fracture. Adjusted multivariate regression analysis revealed that hand grip strength below the cutoff point for sarcopenia according to the EWGSOP2 was an independent predictor of worse functional outcome at 3 and 6 months after hip fracture for both genders and in all age populations.

**Table 2:** Univariate and multivariate analysis for variables significantly associated with Barthel index scores 3 months after fracture.

Predictors	Univariate analysis		Multivariate analysis	
	B (95% CI)	p value	B (95% CI)	p value
**Marital status**	-0.05 (-11.04–5.71)	0.531		
**Preinjury residence**	-0.18 (-17.22 - -1.41)	0.021	-0.13 (-13.29 - -0.34)	0.039
**BMI**	0.02 (-0.79–1.08)	0.761		
**CCI**	-0.29 (-15.40 - -5.24)	<0.001	-0.10 (-8.12–1.06)	0.131
**SPMSQ**	0.27 (1.72–5.97)	<0.001	0.72 (-0.92–2.94)	0.302
**EQ5D before fracture**	0.31 (25.94–71.55)	<0.001	0.16 (2.10–46.10)	0.032
**Barthel index before fracture**	0.51 (1.37–2.30)	<0.001	0.36 (0.77–1.71)	<0.001
**HGS**	0.31 (8.29–24.51)	<0.001	0.185 (2.29–16.71)	0.010
**Time from admission to operation**	-0.01 (-1.36–1.13)	0.860		
**Lenght of hospital stay**	-0.04 (-1.04–0.62)	0.616		
**Type of anesthesia**	0.09 (-3.22–12.55)	0.244		
**Duration of anesthesia**	-0.21 (-0.31–0.05)	0.009	-0.12 (-0.20–0.01)	0.069
**Complications**	-0.16 (-18.58 - -0.44)	0.040	-0.13 (-14.90 - -0.20)	0.044

Adjusted for age and gender

BMI—body mass index; CCI—Charlson Comorbidity Index; SPMSQ—Short Portable Mental Status Questionnaire; HGS—handgrip strength

**Table 3:** Univariate and multivariate analysis for variables significantly associated with Barthel index scores 6 months after fracture.

Predictors	Univariate analysis		Multivariate analysis	
	B (95% CI)	p value	B RR (95% CI)	p value
**Marital status**	-0.12 (-14.75–2.56)	0.166		
**Preinjury residence**	-0.14 (-15.52–1.20)	0.093		
**BMI**	0.04 (-0.75–1.28)	0.602		
**CCI**	-0.31 (-16.61 - -5.58)	<0.001	-0.17 (-10.99 - -1.03)	0.018
**SPMSQ**	0.29 (1.84–6.30)	<0.001	0.09 (-0.76–3.26)	0.222
**EQ5D before fracture**	0.36 (32.11–78.99)	<0.001	0.15 (-0.28–45.56)	0.053
**Barthel index before fracture**	0.53 (1.47–2.48)	<0.001	0.38 (0.86–1.91)	<0.001
**HGS**	0.36 (10.39–27.18)	<0.001	0.21 (3.07–18.37)	0.006
**Time from admission to operation**	-0.07 (-1.87–0.74)	0.392		
**Lenght of hospital stay**	0.01 (-0.83–0.94)	0.895		
**Type of anesthesia**	0.08 (-4.19–12.28)	0.333		
**Duration of anesthesia**	-0.17 (-0.28 - -0.01)	0.037	-0.08 (-0.17–0.05)	0.250
**Complications**	-0.07 (-14.06–5.82)	0.414		

Adjusted for age and gender

BMI—body mass index; CCI—Charlson Comorbidity Index; SPMSQ—Short Portable Mental Status Questionnaire; HGS—handgrip strength

Besides hand grip strength, living at home, better quality of life and higher functionally independence before fracture, as well as absence of complications during the hospitalization period were independent predictors of Barthel index scores 3 months postoperatively (Table 2). Multivariate regression analysis showed that, besides hand grip strength above cutoff values for sarcopenia, lower CCI index and higher Barthel index scores before fracture were independent predictors of higher Barthel index scores 6 months after fracture (Table 3).

## Discussion

Our results showed that 35% of the study population had relevant clinical weakness based on hand grip strength. Patients with weak grip strength were of older age, had a higher level of comorbidity, lower cognitive level, lower functional level, and worse quality of life at admission. This clearly indicates a decline of reserve and function across multiple physiological systems in this group of patients.

Our study demonstrates that hip fracture patients with a validated threshold for clinical weak grip strength assessed at an early stage had significantly poorer functional recovery after 3 and 6 months compared to patients with a grip strength above the cutoff points. Furthermore, our findings provide evidence that hand grip strength along with several other prognostic factors, such as age, pre-injury residence, functional status and health-related quality of life, presence of comorbidities, and postoperative complications, traditionally considered in clinical practice can independently predict short- and long-term functional outcome [30].

The results of our investigation are consistent with data from previous studies confirming the prognostic role of handgrip strength after hip fracture [15–21]. However, it is not easy to compare present results with other studies addressing this subject. First, handgrip strength was assessed at various time points in different studies. There are only several studies evaluating the prognostic value of handgrip strength measured in the acute setting after hip fracture [19, 20, 31]. Savino et al. showed that handgrip strength measured at hospital admission significantly predicted walking recovery 12 months after hip fracture [19]. Alvarez MN et al. concluded that HGS asessed in the first hours after hospital admission for hip fracture surgery is an indicator of functional recovery after three months [20]. Menéndez-Colino et al. also assessed grip strength 72h upon hospital admission. They showed that low hand-grip strength is associated with long-term mortality, and did not investigate its relation to functional outcome [31]. Wehren et al. found that handgrip strength predicted the self-reported ability to function in activities of daily living during a 12-month follow-up period [15]. All the other studies assessed grip strength at a later time point after hip fracture. Beloosesky et al. showed that handgrip strength was significantly associated with functional independence 6 months after hip fracture [17]. Di Monaco et al. reported a significant association between grip strength at admission to a rehabilitation hospital and functional outcome at the end of inpatient rehabilitation [16, 21], and at a 6-month follow-up [21]. Second, a small body of literature used cutoff points to define clinical relevant weakness based on HGS [5, 18, 20], and no study applied the EWGSOP2 criteria. Alvarez MN et al. [20] who used the EWGSOP criteria [4], and Di Monaco et al. [18] who used the FNIH Sarcopenia Project criteria for HGS categorization [32] confirmed the prognostic role of hand grip strength. In contrast, Steihaug et al. who applied the EWGSOP criteria to investigate the impact of HGS early after fracture were the only ones who found no association between grip strength and short- and long-term functional outcome [5]. This is the only study to deny the value of HGS in predicting functional outocome in hip fracture patients. It has to be taken into account that although the definition of „weakness”according to the FNIH, and the EWGSOP criteria correspond very closely to the one defined by the EWGSOP2 [3], it is not the same. Moreover, our results cannot be compared completely to those published by Di Monaco et al., because they reported their results only on women, and in the postacute rehabilitation setting [16, 18].

There are several strengths of our study. First, to the best of our knowledge this is the first study to assess HGS using the EWGSOP2 criteria. Second, our study proves the prognostic value of HGS in the acute setting for both gender and all ages. Most studies who analyzed the predictive role of hand grip strength reported their results only on women with hip fracture [15, 16, 18]. There are also some limitations to our study. First, the outcome of our study was assessed with only self-reported information collected by phone interviews. Second, patients were collected only from one single center. Additionally, there are other confounding factors that could have been studied, for example nutritional status and vitamin D status. We are also completely aware of the fact that our hand grip strength measurement protocol did not adhere to the standardized method advocated by the American Society of Hand Therapists (ASHT), where the subjects are tested in a seated position [33]. Grip strength was measured prior to surgery in the supine position, because we wanted to evaluate the impact of baseline characteristics of our patients on functional recovery. Measuring grip strength after surgery in a seated position would be definitely a more standardized way to assess muscle strength. However, measuring grip strength upon admission in patients with hip fracture in the acute setting is the only way to investigate its predictive value as a baseline variable. It is well known that muscle mass is maintained during the first 10 days after hip fracture, although it subsequently diminishes [34, 35]. Consequently, it is reasonable to assume that measuring HGS early after hip fracture is an appropriate time to assess function. This approach was also used by other authors [19, 20, 31].

Our results have two clinical implications. First, there is an ongoing urgent need to identify hip fracture patients at increased risk for worse outcome. Recognizing this vulnerable group of patients allows for initiation of prevention and specific intervention to avoid the debilitating consequences of hip fracture. Gait speed and muscle mass cannot be assessed before surgery. Thus, functional evaluation at this stage is limited to measuring muscle strength. Confirmation of the prognostic value of HGS assessed in the acute setting is therefore very significant. Second, muscle weakness is a modifiable risk factor that can be improved. It is well know that strengthening exercises had favorable effects on various outcomes after hip fracture [36]. Thus, early assessment of HGS among older hip fracture patients can contribute to the development of individualized treatment plans aimed at improving functional outcomes. Therefore, future studies should reveal if patients with clinically defined weakness who sustain a hip fracture could benefit from prompt interventions to improve muscle strength, function, and outcomes. Furthermore, sample size was not calculated at the beginning of the research. Using G Power 3.1.9.2 [37], posthoc achieved study power was calculated for effect size of 0.3, error of the first type 0.05 and the total number of respondents with hip fracture n = 191. The calculated study power equals 98.56%, which indicates good study power.

Our study has identified HGS assessed in the acute setting as potential prognostic predictor of functional outcome in patients with hip fracture. Hand grip strength is an accessible, cost effective, and simple objective measure of physical function for bedridden patients. Thus, clinicians should be encouraged to include hand grip assessment in their evaluation of hip fracture patients at admission to the acute setting in order to optimize prognostic counseling and treatment of high-risk individuals. Further studies are needed to investigate the relevance of early introduction of resistance exercise programs in hip fracture patients with relevant low muscle strength.

## Supporting information

S1 File Dataset. (XLSX)

## References

[1] Bandeen-Roche K, Xue QL, Ferrucci L, Walston J, Guralnik JM, Chaves P, et al Phenotype of frailty: characterization in the women's health and aging studies. J Gerontol A Biol Sci Med Sci. 2006 3;61(3):262–6. 10.1093/gerona/61.3.262 16567375

[2] Fielding RA, Vellas B, Evans WJ, Bhasin S, Morley JE, Newman AB, et al Sarcopenia: an undiagnosed condition in older adults. Current consensus definition: prevalence, etiology, and consequences. International working group on sarcopenia. J Am Med Dir Assoc. 2011 5;12(4):249–56. 10.1016/j.jamda.2011.01.003 21527165 PMC3377163

[3] Cruz-Jentoft AJ, Bahat G, Bauer J, Boirie Y, Bruyère O, Cederholm T, et al Sarcopenia: revised European consensus on definition and diagnosis. Age Ageing. 2019 1 1;48(1):16–31. 10.1093/ageing/afy169 30312372 PMC6322506

[4] Cruz-Jentoft AJ, Baeyens JP, Bauer JM, Boirie Y, Cederholm T, Landi F, et al Sarcopenia: European consensus on definition and diagnosis: Report of the European Working Group on Sarcopenia in Older People. Age Ageing. 2010 7;39(4):412–23. 10.1093/ageing/afq034 20392703 PMC2886201

[5] Steihaug OM, Gjesdal CG, Bogen B, Kristoffersen MH, Lien G, Ranhoff AH. Sarcopenia in patients with hip fracture: A multicenter cross-sectional study. PLoS One. 2017 9 13;12(9):e0184780 10.1371/journal.pone.0184780 28902873 PMC5597226

[6] Landi F, Calvani R, Ortolani E, Salini S, Martone AM, Santoro L, et al The association between sarcopenia and functional outcomes among older patients with hip fracture undergoing in-hospital rehabilitation. Osteoporos Int. 2017 5;28(5):1569–1576. 10.1007/s00198-017-3929-z 28154941

[7] He H, Liu Y, Tian Q, Papasian CJ, Hu T, Deng HW. Relationship of sarcopenia and body composition with osteoporosis. Osteoporos Int. 2016 2;27(2):473–82. 10.1007/s00198-015-3241-8 26243357

[8] Oliveira A, Vaz C. The role of sarcopenia in the risk of osteoporotic hip fracture. Clin Rheumatol. 2015 10;34(10):1673–80. 10.1007/s10067-015-2943-9 25912213

[9] Tarantino U, Piccirilli E, Fantini M, Baldi J, Gasbarra E, Bei R. Sarcopenia and fragility fractures: molecular and clinical evidence of the bone-muscle interaction. J Bone Joint Surg Am. 2015 3 4;97(5):429–37. 10.2106/JBJS.N.00648 25740034

[10] Benichou O, Lord SR. Rationale for Strengthening Muscle to Prevent Falls and Fractures: A Review of the Evidence. Calcif Tissue Int. 2016 6;98(6):531–45. 10.1007/s00223-016-0107-9 26847435

[11] Hirschfeld HP, Kinsella R, Duque G. Osteosarcopenia: where bone, muscle, and fat collide. Osteoporos Int. 2017 10;28(10):2781–2790. 10.1007/s00198-017-4151-8 28733716

[12] Rantanen T, Volpato S, Ferrucci L, Heikkinen E, Fried LP, Guralnik JM. Handgrip strength and cause-specific and total mortality in older disabled women: exploring the mechanism. J Am Geriatr Soc. 2003 5;51(5):636–41. 10.1034/j.1600-0579.2003.00207.x 12752838

[13] Syddall H, Cooper C, Martin F, Briggs R, Aihie Sayer A. Is grip strength a useful single marker of frailty? Age Ageing. 2003 11;32(6):650–6. 10.1093/ageing/afg111 14600007

[14] Chen LK, Liu LK, Woo J, Assantachai P, Auyeung TW, Bahyah KS, et al Sarcopenia in Asia: consensus report of the Asian Working Group for Sarcopenia. J Am Med Dir Assoc. 2014 2;15(2):95–101. 10.1016/j.jamda.2013.11.025 24461239

[15] Wehren LE, Hawkes WG, Hebel JR, Orwig DL, Magaziner J. Bone mineral density, soft tissue body composition, strength, and functioning after hip fracture. J Gerontol A Biol Sci Med Sci. 2005 1;60(1):80–4. 10.1093/gerona/60.1.80 15741287

[16] Di Monaco M, Castiglioni C, De Toma E, Gardin L, Giordano S, Di Monaco R, et al Handgrip strength but not appendicular lean mass is an independent predictor of functional outcome in hip-fracture women: a short-term prospective study. Arch Phys Med Rehabil. 2014 9;95(9):1719–24. 10.1016/j.apmr.2014.04.003 24769122

[17] Beloosesky Y, Weiss A, Manasian M, Salai M. Handgrip strength of the elderly after hip fracture repair correlates with functional outcome. Disabil Rehabil. 2010;32(5):367–73. 10.3109/09638280903168499 20025431

[18] Di Monaco M, Castiglioni C. Weakness and Low Lean Mass in Women With Hip Fracture: Prevalence According to the FNIH Criteria and Association With the Short-Term Functional Recovery. J Geriatr Phys Ther. 2017 Apr-Jun;40(2):80–85. 10.1519/JPT.0000000000000075 26703524

[19] Savino E, Martini E, Lauretani F, Pioli G, Zagatti AM, Frondini C, et al Handgrip strength predicts persistent walking recovery after hip fracture surgery. Am J Med. 2013 12;126(12):1068–75.e1. 10.1016/j.amjmed.2013.04.017 24054175

[20] Alvarez MN, Bonnardeaux P L.D, Thuissard IJ, Sanz-Rosa D, Muñana EA, Galindo RB, et al Grip strength and functional recovery after hip fracture: An observational study in elderly population. Eur Geriatr Med. 2016 12 1;7(6):556–60.

[21] Di Monaco M, Castiglioni C, De Toma E, Gardin L, Giordano S, Tappero R. Handgrip strength is an independent predictor of functional outcome in hip-fracture women: a prospective study with 6-month follow-up. Medicine (Baltimore). 2015 2;94(6):e542. 25674760 10.1097/MD.0000000000000542 PMC4602757

[22] Pfeiffer E. A short portable mental status questionnaire for the assessment of organic brain deficit in elderly patients. J Am Geriatr Soc. 1975 10;23(10):433–41. 10.1111/j.1532-5415.1975.tb00927.x 1159263

[23] Beaton DE, O'Driscoll SW, Richards RR. Grip strength testing using the BTE work simulator and the Jamar dynamometer: a comparative study. Baltimore Therapeutic Equipment. J Hand Surg Am. 1995 3;20(2):293–8. 7775773 10.1016/s0363-5023(05)80029-2

[24] Richards LG. Posture effects on grip strength. Arch Phys Med Rehabil. 1997 10;78(10):1154–6. 9339168 10.1016/s0003-9993(97)90143-x

[25] Katz S, Ford AB, Moskowitz RW, Jackson BA, Jaffe MW. Studies of Illness in the Aged. The Index of ADL: A Standardized Measure of Biological and Psychological Function. JAMA. 1963 9 21;185:914–9. 10.1001/jama.1963.03060120024016 14044222

[26] Mahoney FI, Barthel D. Functional evaluation: The Barthel Index. Md State Med J. 1965 2;14:61–5. 14258950

[27] Brooks R. EuroQol: the current state of play. Health Policy. 1996 7;37(1):53–72. 10158943 10.1016/0168-8510(96)00822-6

[28] Dolan P. Modeling valuations for EuroQol health states. Med Care. 1997 11;35(11):1095–108. 9366889 10.1097/00005650-199711000-00002

[29] Charlson ME, Pompei P, Ales KL, MacKenzie CR. A new method of classifying prognostic comorbidity in longitudinal studies: development and validation. J Chronic Dis. 1987;40(5):373–83. 3558716 10.1016/0021-9681(87)90171-8

[30] Kristensen MT. Factors affecting functional prognosis of patients with hip fracture. Eur J Phys Rehabil Med. 2011 6;47(2):257–64. 21597435

[31] Menendez-Colino R, Alarcon T, Gotor P, Queipo R, Ramirez-Martin R, Otero A, et al Baseline and pre-operative 1-year mortality risk factors in a cohort of 509 hip fracture patients consecutively admitted to a co-managed orthogeriatric unit (FONDA Cohort). Injury. 2018 3;49(3):656–661. 10.1016/j.injury.2018.01.003 29329713

[32] Alley DE, Shardell MD, Peters KW, McLean RR, Dam TT, Kenny AM, et al Grip strength cutpoints for the identification of clinically relevant weakness. J Gerontol A Biol Sci Med Sci. 2014 5;69(5):559–66. 10.1093/gerona/glu011 24737558 PMC3991145

[33] Fess E.E. Grip strength In: Casanova J.S., editor. Clinical assessment recommendations. 2nd ed. The American Society of Hand Therapists; Chicago (IL): 1992 pp. 41–45.

[34] D'Adamo CR, Hawkes WG, Miller RR, Jones M, Hochberg M, Yu-Yahiro J, et al Short-term changes in body composition after surgical repair of hip fracture. Age Ageing. 2014 3;43(2):275–80. 10.1093/ageing/aft198 24370941 PMC3927774

[35] Fox KM, Magaziner J, Hawkes WG, Yu-Yahiro J, Hebel JR, Zimmerman SI, et al Loss of bone density and lean body mass after hip fracture. Osteoporos Int. 2000;11(1):31–5. 10.1007/s001980050003 10663356

[36] Lee SY, Yoon BH, Beom J, Ha YC, Lim JY. Effect of Lower-Limb Progressive Resistance Exercise After Hip Fracture Surgery: A Systematic Review and Meta-Analysis of Randomized Controlled Studies. J Am Med Dir Assoc. 2017 12 1;18(12):1096.e19–1096.e26. 10.1016/j.jamda.2017.08.021 29033325

[37] Available from: http://www.gpower.hhu.de/

